# An Inquiry into the Proximate Cause of Gout and Its Rational Treatment

**Published:** 1852-09

**Authors:** Anthony White

**Affiliations:** Cambridge, late President of the Royal College of Surgeons of England


### RECORD OF MEDICAL SCIENCE.

### PATHOLOGY AND PRACTICE OF MEDICINE.

*An Inquiry into the proximate cause of Gout and its rational Treatment.* By ANTHONY WHITE, Esq., M. B., Cambridge, late President of the Royal College of Surgeons of England.—I have for some time been engaged in preparing a work on Diet, wherein I purpose, among other things, to trace out the connection between sundry constitutional disorders, and the habitual abuses of the digestive organs in childhood as well as in the adult age. I had intended to embody in that work certain theoretical and practical views, which long experience and reflection have led me to entertain on the subject of gout; but having been strongly solicited by several professional friends not to delay the publication of that portion of my notes, I have here thrown them into the shape of a separate paper.

In venturing to propound a new theory of gout, I do not conceal from myself the hazards I incur. The very announcement of my design must, I am aware, provoke against it a formidable array of prejudice, since it is natural to predict the failure of every fresh adventurer in an enterprise so often and so strenuously essayed, and always essayed in vain. On the other hand, I submit that there is a wide distinction between what is merely improbable and what is impossible, and that, however difficult be the problem I profess to solve, at least, it involves no absolute impracticability. It is safe to reject, *à priori*, the claims of one who shall pretend to have discovered the perpetual motion, or the elixir vitæ, or to have unravelled the impenetrable mysteries of ontology; but an inquiry into the natural history of any given disease belongs to quite another category; nor does there exist any reason why science should ever halt in despair at any unaccomplished point in her proper business, which is in every instance to trace back step by step those trains of phenomena to which, as we regard them in their unvaried order of sequence, we attribute the relationship of cause and effect. In some cases, this kind of research has been prosecuted almost to its last limits, whilst in others it has stopped short at an early stage, and there remained for centuries, in spite of countless efforts to discover the missing clue to the next step. But soon or late the clue will be found, and the further step achieved; for no amount of lost labor can exhaust the persevering energies of science—no lapse of time can subject her powers to bar or proscription. How often—to use the language of Sir John Herschel—how often have "we seen obscurities, which seemed impenetrable, in physical and mathematical science, suddenly dispelled, and the most barren and unpromising fields of inquiry converted, as if by inspiration, into rich and inexhaustible springs of knowledge and power, on a simple change of our point of view, or by merely bringing to bear on them some principle that it had never occurred before to try?"

I believe that, without arrogating to myself any inordinate share of acumen, I may affirm that, through one of those happy accidents adverted to in the foregoing extract, I have been prompted to the true answer to that hitherto unsolved question—What is the proximate cause of gout?

In addition to the ordinary opportunities of a long professional life, my means of becoming intimately acquainted with this disease have been in part of a peculiar nature, such as falls in an equal degree to the lot of few medical practitioners, and such, I may boldly assert, as no man will be inclined to envy me. Corvisart's classical treatise on diseases of the heart was the work of a man who was himself afflicted with one of those organic maladies he so ably described. The symptoms of ulceration of the stomach were vividly portrayed by Béclard, from his own sad personal experience. The connection between organic disease of the brain, and certain disorders of the sensorial functions was illustrated, as it could never otherwise have been, by Dr. Wollaston's description of his own case, which he studied with the same serene sagacity and precision as characterized every other exertion of his noble intellect. I, too, however unfitted to compare in other respects with those illustrious men, have this, at least, in common with them, that I have learned from my own sufferings some facts likely, as I trust, to prove of considerable importance to medical science.

I am the offspring of parents both of whom were constantly the subjects of gout—a disease which was inherited by their four sons. Two of the latter (twins) died at the respective ages of 45 and 46, worn out by reiterated attacks of the malady. For myself, sharing largely in the family predisposition, I very early in life began to exhibit signs of latent gout, shown in the ready conversion of common nutriment into acrid acidity; and among my earliest recollections are my mother's repeated administrations to me of magnesia and alkaline preparations, to remedy the heartburn, with which I was constantly tormented. About the age of sixteen, a fixed aching pain occupied the middle flexor tendon of my right hand near the root of the finger, preventing its flexure. In the course of a week or two, the pain in the finger ceased suddenly, and was almost immediately succeeded by a severe attack of gout in the large joint of the great toe, ushered in by all the usual precursory symptoms. The subsequent visitations of the disease have extended over a period of forty years, during which it has affected every tissue of my body. Hence, I have had abundant opportunity not only to experiment upon the gout in my own person as regards dietetics and therapeutics, but also to study its natural history under the least ambiguous conditions, whenever, as not unfrequently happened, I allowed a paroxysm to run its course, and effect its own cure. It was chiefly by noticing what took place under such circumstances that I was led to entertain those views which I shall presently lay before my reader.

But first, for the sake of clearness, it will be well to define the actual state of our knowledge as to the intimate nature of gout; and this, I think, cannot be better expressed than in the following propositions wherein Dr. Holland has comprised all that is ascertained, or to be strongly presumed, on the subject:

1. "That there is some part of bodily organization disposing to gout, because it is an hereditary disease.

2. "That there is a *materies morbi*, whatever its nature, capable of accumulation in the system, of change of place within the body, and of removal from it.

3. "That though identity be not hitherto proved, there is a presumable relation between the lithic acid, or its compounds, and the matter of gout; and a connection through this with other forms of the calculous diathesis.

4. "That the accumulation of this matter of the disease may be presumed to be in the blood; and its retrocession or change of place, when occurring, to be effected through the same medium.

5. "That an attack of gout, so called, consists in, or tends to produce the removal of this matter from the circulation, either by deposition in the parts affected by the excretions, or in some other less obvious way, through the train of actions forming the paroxysm of the disorder.

6. "That there is an intimate relation between the condition of gouty habit and the functions of the kidneys and liver, both in health and disease.

7. "And that the same state of habit or predisposition which in some persons produces the outward attack of gout, does in others, and particularly in females, testify itself solely by disorder of internal parts, and especially of the digestive organs."

The opinion that hereditary predisposition to gout consists solely in a peculiar character of the ligamentous and other associated textures, is surely untenable, although it has been advocated by some authors of eminence. The disease, however prone to affect the joints chiefly, is incident likewise to all the other fibrous textures of the body without exception. The constitutional disturbance that precedes its attacks, the many functional aberrations of the assimilating, secretory, and excretory organs by which it is accompanied,—its erratic character, and the rapidity of its transitions from one part to another,—are facts tending most strongly to the conclusion that the immediate cause of the malady is not local, but general, and that the vehicle of its diffusion over the whole system can be nothing else than the circulating fluids.

Furthermore, did we suppose that hereditary transmission of gout is identified with a peculiar condition of those solids which are the most frequent seat of gouty inflammation, its active development would then have to be accounted for in one or other of the two following ways:—Either the transmitted peculiarity in question is an actual *materies morbi* deposited in the vitiated textures, or it is such a structural peculiarity of the latter as renders them especially liable to the noxious influence of a morbid principle produced in the body by other causes. Either hypothesis leads to the conclusion that gout is a blood disease. The second of the two does this directly and immediately, for it assumes the independent existence of an exciting cause, to be brought in contact with the morbidly predisposed parts through the medium of the circulation; whilst, on the other hypothesis, it is evident that the transmitted *materies morbi* must be taken up into the blood, contaminating its mass, and producing in it effects analogous to those caused by other animal poisons imbibed from without.

But there is another class of solids, namely, those concerned in the functions of organic life, which have paramount claims to attention in every inquiry like the present. It is evident that any inherent vice in one or other of the great chylopoietic viscera, must of necessity induce a proportional depravity in the circulating fluids. Reasoning, then, *à priori*, there is nothing unwarrantable in the conjecture that the real *fons malorum* transmitted by the gouty to their offspring, is an unwholesome blood-making apparatus. Such a conjecture, I repeat, is by no means improbable, and my own observations and reflections are all in favor of its positive truth.

On the whole, then, we may safely admit that hereditary gout is a disposition to generate a certain morbid matter within the body, whether that disposition be the effect of some abnormal organic condition, promoting its formation or impeding its due excretion, or of some transmitted impurity of blood which tends, as usual in such cases, to reproduce and continue itself by vitiating the nutritive functions.

The same disposition, but created by other causes, must obviously exist in those cases in which gout occurs as an idiopathic disease. Its individual or ancestral origin is a circumstance which may influence the intensity of its development and its pertinacity in the system, but in no way affects its intrinsic nature. Whether hereditary or not, it presents the same general characteristics, and is of course attributable to the same material agent.

Setting out, then, from this cardinal principle of a *materies morbi* circulating with the blood, we have next to investigate its nature and its origin. And here we are struck, on the very threshold of the inquiry, by the close affinity between the gouty and the lithic acid diathesis—an affinity so remarkable that a very general disposition prevails among medical writers to consider lithic acid as the true gouty poison, and to impute its presence in the system to the impaired action of the kidneys.

As to this latter notion, the arguments adduced in support of it appear to me to be based on a singular misapprehension of patent facts. The discharge of lithic acid and its salts in the urine is a salutary process; and while the kidneys are actively performing such a process, it is strange indeed to charge them with creating the offensive matter they only serve to remove. It is not from the presence of lithic acid sediments in the urine of the gouty, but from their absence, that we should be warranted in ascribing to defective action of the kidneys the accumulation of that excrementitious matter in the system. If the blood was manifestly surcharged with lithic salts or their equivalents, while none such escaped in the urine, then, indeed, we should have reached the end of our inquiry in full assurance that the kidneys were the very matrices of gout. But it is not so in reality: and the most we can venture to assert is, that the renal functions, in common with others, are secondarily affected by the cause, whatever it be, of the gouty diathesis.

I think it the more necessary to insist on this point, as it is one on which so acute and lucid a reasoner as Dr. Holland appears to have fallen into error. "The kidneys," he says, "are evidently the organs of the body upon the disordered or deficient action of which depend those changes in the circulating fluids which have the closest relation to all the phenomena of gout." He would, I think, have been nearer the truth if he had said that the kidneys are, of all organs, those whose secretions afford the most faithful and the most readily discernible evidence of the changes aforesaid.

However intimate the connection between the gouty and the lithic acid diathesis, evidence is yet wanting to establish their actual identity. If the *materies morbi* we are in search of was nothing else than lithic acid, we should naturally expect to find every considerable development of that product followed by a gouty paroxysm. But this is notoriously not the case. It is no uncommon thing to find the urine constantly loaded, during a long period, with lithic acid sediments, without the occurrence of a single gouty symptom; while, on the other hand, it is known that the gouty paroxysm sometimes occurs without the excess of lithic acid in the urine. Instances of this kind, occurring in æsthenic forms of the disease, have been mentioned by Dr. Todd in the Croonian Lectures for the year 1843:—"I have remarked," he says, "a peculiarity belonging to most of the cases of this kind that I have met with, namely, that the urine does not exhibit the abundant precipitate of lithates which so often accompanies the gouty paroxysm. In some instances there was no precipitate at all; and in others it was very slight. And the specific gravity of the urine was rather below than above the ordinary standard, indicating that no excessive quantity of either urea or lithic acid was held in solution."

The gouty poison, then, is not identical with lithic acid, but is so near akin to it that the chemical and pathological characteristics of the latter may probably yet serve as indices to guide us to the discovery of the former.

"Organic chemistry," says Dr. Holland, "has taught us how readily the elements out of which all animal matter is formed are displaced from one combination and enter into others; and how very slight, frequently, are the differences, indicated by analysis, between substances eminently noxious to the system, and those indifferent or beneficial to it. We owe, further, to recent experiments the explicit proof of what simple observation had partly shown before—the remarkable effect upon the whole mass of the blood of minute quantities of certain matters brought into the circulation—leading to the inference of analogous effects from an increased proportion of one or other of the principles accumulating or being unduly retained in the body. . . . . These circumstances, now familiar to us, do certainly not identify the material cause of gout with any of the animal excretions just named [lithic acid, urea, the lithic or purpuric salts, &c.]; but they tend to concentrate our views towards them, and give a much more specific direction to future research. The assured connection of the gouty with the calculous diathesis,—the chemical nature of the concretions and deposits in the former,—and the evidence that these deposits often become in part a substitute for the more active forms of the disease—all concur in further sanctioning the same general view. If we cannot affirm that urea, the lithic acid, or other animal compounds circulating in the blood, give cause to the phenomena of gout, under the most cautious reasoning, we are at least entitled to assume, with some confidence, that these matters secreted from the kidneys *are the equivalents to gouty matter present in the system,*—that they have certain proportion of quantity to each other,—and that upon their balance depend all the essential characters of the disease,—its modifications being determined by various causes; some of them topical, some belonging to general functions implicated in the effects of this common cause."

I particularly invite the reader's attention to the words above printed in italics. They imply that the morbid developments of lithic acid and its salts may be due to the presence of some principle altogether unlike them in sensible properties and chemical composition.

And now we may proceed to deal with the special object of this paper, which aims at determining the primary seat and the essential nature of the disease in question. To this end I shall succinctly narrate the course of induction whereby I arrived at those views which I desire to recommend to the candid examination of my professional brethren.

Having endured innumerable visitations of gout, and having had recourse to a variety of medicaments, some of which were fearfully destructive to my general health, I at last set about watching attentively the method which nature herself adopts for the cure of this disease. Thus it frequently happened, during my forty years' conflict with my hereditary malady, that I submitted to the old plan of patience and flannel, leaving the disorder to run its course and wear itself out by its own violence. On several of these occasions I was attacked with sickness and vomiting, accompanied by acrid bilious discharges from the bowels; and these evacuations were followed by immediate relief as to every local and constitutional symptom. Sometimes the result was an entire cessation of the paroxysm; at other times the alleviation was more partial; but repeated experience convinced me that the degree of relief obtained was always proportioned to the copiousness of the bilious evacuations. Pursuing this hint given me by nature, when the spontaneous diarrhœa has been too scanty I have assisted it with five grains of calomel. These in a few hours produced copious bilious discharges; the gout departed, and I was well again.

The conclusion forced upon my mind by these facts, recurring again and again during a period of so many years, is, that not to the stomach or the kidneys, or to the impaired functions of any other viscus than the liver, is the cause of gout ascribable.

In corroboration of this view I may appeal to the character of all those medicaments which at various times have been held in estimation as specifics against gout. One property is common to them all—namely, that of strongly stimulating the hepatic functions. The *eau médicinale,* which was introduced into this country about twenty years ago from France, was a remedy of this class. It was sold in one-drachm bottles (this was the dose), and its effects were certainly very remarkable—frequently removing the most painful attacks of gout in one night. The composition of this potent nostrum long remained a secret; it was conjectured to contain white hellebore; and I recollect the physicians of the Westminster Hospital prescribing a vinous infusion of the latter, in one-drachm doses, with great success, as a substitute for the eau médicinale. The revived use of colchicum or meadow saffron, which I believe to be the essential ingredient in the eau médicinale, has put us into possession of an invaluable antidote to gout; but how does this colchicum act beneficially? Assuredly not on the stomach, which it nauseates; assuredly not on the heart or circulation, which it distresses: but it acts on the secretions of the liver; and long personal experience has taught me that until the functions of that organ are called into vigorous play, the colchicum is worse than useless.

Latterly it has been my practice to use colchicum in combination with other medicines. When I was in the habit of taking it singly, my dose was generally about sixty drops of the wine of the seeds, repeated every six hours. After three or four such doses the bowels were acted on; the evacuations had the odor of colchicum; deeply tinted scalding bile was passed, and I was well, for I needed no more.

Now, if a spontaneous evacuation of bile operates critically to the relief of the gouty paroxysm; if five grains of calomel produce relief; if just so much colchicum or other medicine produces relief as is sufficient to cause a copious discharge of bile, then is it demonstrated that the diminished or altered state of the hepatic secretion, which is always a concomitant of gout, is not to be classed among the secondary phe nomena of that disease, as pathologists have hitherto invariably supposed.

Let A and B be any two phenomena whatever; and suppose that B is never found except in company with A; then will there be reason for concluding either that one of the two is the cause of the other, A of B, or B of A, or else that both are parallel effects of some third principle. But suppose it be found that, whereas B never presents itself unaccompanied by A, yet A may exist without B, and that, when both are present, the removal of the former is invariably followed by the disappearance of the latter, then it will be manifest that A is the cause of B.

The correctness of this abstract reasoning will, I presume, be admitted without question. To apply it to the subject of our present inquiry, we have only to substitute for A and B, the phrases "impaired functions of the liver," and "paroxysm of gout."

No writer that I am aware of has ever propounded, or even surmised, the doctrine that the proximate cause of gout is a functional disorder of the liver; and I cannot overcome the astonishment that possesses me when I think that it should have been reserved for me to make such a discovery. The principle, when once divulged, appears so plain and obvious, that it is wonderful it should have been overlooked so long. Such has been the feeling expressed by several of my professional brethren to whom I have communicated my views. Seldom have my conclusions failed in such instances to receive a prompt and full assent, and to elicit from each of my hearers the exclamation, "How is it possible I never thought of that before." But the history of science is full of examples, showing how inquirers have for ages been shut out by the filmiest barriers from the acquisition of precious truths.

The derangement of the liver which always accompanies the gouty paroxysm, and manifests itself by unequivocal signs, such, for instance, as the pale color of the fæces, is too obvious to have escaped notice. Accordingly, writers on the disease have constantly adverted, more or less prominently, to this pathological fact; but they have all failed to assign to it the position it really occupies in the train of symptoms. The tendency of their speculations has generally been to consider the disorder of the liver as consequent upon that of the stomach, whereas the converse doctrine is far more consonant with observation and with physiological principles. Acidity in the stomach is an unfailing element in the gouty diathesis. Now such a condition of that organ may, undoubtedly, react on the liver, and impede or vitiate its secretions. On the other hand, we know that a very important office performed by bile is the neutralization of the free acid, which is always developed in the stomach during healthy digestion, and is, therefore, a constant ingredient in chyme; only assuming a morbid character when it is excessive or otherwise abnormal. Hence, given two coexisting facts—acidity of stomach, and deficiency or faulty composition of bile—it will be natural to surmise that the former is the effect of the latter, and nothing less than specific proof could justify our adoption of the opposite conclusion.

It is a fact of great importance to the decision of this question, that, however the administration of antacid medicines may alleviate the heartburn and the other distressing effects of acidity in the primæ viæ, such remedies never rise above the rank of palliatives in the treatment of gout. They have not the least efficacy in restoring the healthy action of the liver; whilst, on the other hand, whatever accomplishes that object never fails to remove every other dyspeptic symptom likewise.

The liver, then, is the *officina* in which is elaborated the *materies morbi*, on which the whole train of gouty symptoms are dependent. What may be the precise nature of that poison I do not pretend to determine. That remains an interesting subject for future inquiry, to which I may venture to hope that I have given a fresh impulse and an increased prospect of success, by defining its proper point of departure, and the direction it should take. The one new leading fact which I affirm as demonstrated, is sufficient to indicate very distinctly the mode of treatment which offers the only rational hope of removing the gouty diathesis, and also to explain the success which has partially attended the various empirical methods which have been adopted for the cure of the disease.

The main object to be pursued towards the effectual cure of the gouty paroxysm, by the removal of its immediate cause, is the restoration of the natural functions of the liver, as indicated by a copious discharge of bile through the bowels. This object may be attained, more or less promptly and sufficiently, by the administration, either of calomel or colchicum, or of some other potent deobstruent of the hepatic system. But here, as in other instances familiar to the minds of my readers, the principle of combining analogous remedies will be found strikingly advantageous. My own practice has long been to rely exclusively for the cure of gout on the following prescription, the effects of which should be carefully watched by the medical attendant:—

℞. Hydr. Chlorid.
Ext. Colchici Acet.
Ext. Aloes purificati.
Pulv. Ipecac. aa. gr. j.
M. et fiat pilula quartis horis sumenda.

Two or three of such pills are generally enough to produce a considerable disgorgement of the liver, which I then assist with one or two doses of the compound decoction of aloes. By this time the gouty paroxysm has either ceased, or there is a marked subsidence of all its distressing symptoms. The pills may then be administered at longer intervals, varying from eight to twenty-four hours, according to circumstances.

The treatment I have above described possesses the cardinal and paramount requisite of being effectual to the end proposed. In addition to this, it is important to know that the combination of calomel and aloes with colchicum, while quickening and corroborating the specific action of the latter on the liver, seems also to neutralize all the noxious properties of that hitherto formidable medicine.

In conclusion, I repeat, that what is called a fit of gout, is only a peculiar manifestation of a functional disorder of the liver; and that whatever brings about a free evacuation of bile puts an end to the gouty paroxysm.

Having assumed, in the above tract, that the cause of gout is the effect of poison generated *eventually* in the liver by an imperfect assimilation of food, or indigestion, I cannot refrain borrowing from my highly learned and intellectual friend Dr. Watson, an observation which I take the liberty of quoting from his "Lectures on the Practice of Physic," vol. ii., p. 641 :—"I need not remind you of the various ways in which extraneous matters find entrance into the blood. Poisons, under their proper shape and name; medicines, which, misapplied, become poisons; our natural food and drink, which the folly of man converts into poison; the products or dregs of the secondary assimilative process; these are common sources of impurities, more or less hurtful, which mix and circulate with the vital fluid."

